# 

**Published:** 1856-06

**Authors:** 


### CONTENTS.

ORIGINAL COMMUNICATIONS— PAGE.

Glycerine. Inaugural Thesis presented to the Faculty of Rush Medical College, Feb. 1st, 1856. By H. Wardner.......... 247

Forceps versus Embryulcia Instruments. By J. R. Freese, M.D., Bloomington, Ill.......... 252

Case of Paralysis. By S. O. Long, of Big Rock, Ill.......... 256

Ninth Annual Session of the American Medical Association.......... 259

EDITORIAL,.......... 289

